# Targeting the Small Airways with Inhaled Corticosteroid/Long-Acting Beta Agonist Dry Powder Inhalers: A Functional Respiratory Imaging Study

**DOI:** 10.1089/jamp.2020.1618

**Published:** 2021-09-27

**Authors:** Henrik Watz, Sara Barile, Daniele Guastalla, Eva Topole, Daniela Cocconi, Benjamin Mignot, Dennis Belmans, Dildar Duman, Gianluigi Poli, Leonardo M. Fabbri

**Affiliations:** ^1^ Pulmonary Research Institute at Lungen Clinic Grosshansdorf, Airway Research Centre North (ARCN), German Centre for Lung Research (DZL), Grosshansdorf, Germany.; ^2^ Chiesi Farmaceutici, S.p.A., Parma, Italy.; ^3^ FLUIDDA, Kontich, Belgium.; ^4^ Department of Pulmonology, Süreyyapaşa Chest Diseases Training and Research Hospital, Istanbul, Turkey.; ^5^ Department of Respiratory and Internal Medicine, University of Ferrara, Ferrara, Italy.

**Keywords:** dry powder inhaler, functional respiratory imaging, inhaled corticosteroid, long-acting beta_2_-agonist, lung deposition

## Abstract

***Background:*** Peripheral deposition of inhaled medication is important as small airway disease has a key role in asthma. In this study, we compared the lung deposition at different mean flow rates of three inhaled corticosteroid (ICS)/long-acting beta_2_-agonist (LABA) combinations delivered by dry powder inhaler (DPI), that is, Foster NEXThaler^®^ (extrafine formulation of beclomethasone/formoterol), Relvar Ellipta^®^ (fluticasone furoate/vilanterol trifenatate), and Symbicort Turbohaler^®^ (budesonide/formoterol).

***Materials and Methods:*** *In vitro* drug delivery parameters were applied to lung computerized tomography (CT) scans of 20 asthma patients by functional respiratory imaging (FRI). Aerosol airway deposition patterns were calculated as percentage (standard deviation) intrathoracic versus extrathoracic deposition, percentage peripheral deposition, and central-to-peripheral (C/P) ratio at different inspiratory mean flow rates.

***Results:*** At 60 and 40 L/min, intrathoracic deposition of ICS/LABA was significantly higher with NEXThaler versus Ellipta. Peripheral deposition (60 L/min) with NEXThaler was higher than Ellipta for ICS (24.7% [3.5%] vs. 5.0% [2.0%]; *p* < 0.001) and LABA (25.3% [3.5%] vs. 13.0% [3.0%]; *p* < 0.001). C/P ratio with NEXThaler was lower (indicating higher peripheral deposition) than Ellipta (ICS: 0.63 vs. 1.63; LABA: 0.63 vs. 0.99). Inspiratory flow rate did not impact lung deposition with NEXThaler or Ellipta. In contrast, Turbohaler performance was negatively impacted by decreasing inspiratory flow rate. In fact, although lung deposition with Turbohaler was similar to that of NEXThaler at 60 L/min, lung deposition with Turbohaler was significantly lower than NEXThaler at both 40 L/min (∼30%) and 30 L/min (∼50%).

***Conclusions:*** Using FRI, we demonstrated better peripheral deposition and C/P ratios of ICS/LABA with NEXThaler versus Ellipta. NEXThaler demonstrated inspiratory flow rate independency of lung deposition versus Turbohaler. These findings suggest that the extrafine formulation is superior to large particle formulations in delivering ICS/LABA consistently both to the large and small airways.

## Introduction

Worldwide, the daily lives of millions of people are affected by asthma.^(1)^ Asthma is a complex disease characterized by chronic inflammation affecting the entire respiratory tract with heterogeneity in clinical presentation.^(1,2)^ Inhalation therapy is the mainstay of asthma treatment with specific benefits, including rapid onset of action, high medication concentrations in the airways and fewer systemic adverse events compared with systemic delivery.^(1)^ Unfortunately, a large proportion of patients still have inadequately controlled asthma with currently available treatments.^(3)^

There is increased recognition of the key role of small airway dysfunction in asthma, and hence the importance of peripheral lung deposition of inhaled medication in asthma control.^(4–7)^ The cross-sectional phase of the ongoing prospective cohort study, the Assessment of Small Airways Involvement in Asthma (ATLANTIS), confirmed the complexity of small airway dysfunction in asthma, with the need for multiple function tests for adequate detection.^(7)^ In addition, ATLANTIS reported that small airway dysfunction occurs across all asthma severities,^(7)^ highlighting the need to target the peripheral lung for optimal asthma control.^(4,6)^

Typically, first-line treatment of asthma is an inhaled corticosteroid (ICS), which can then be combined with second-line treatment of a long-acting beta_2_-agonist (LABA).^(1)^ As small airways have an internal diameter of <2 mm, particle size is critical in ensuring that asthma medication reaches the lung periphery—larger numbers of small particles (<2 μm) are deposited in small airways compared with larger particles.^(4,5,8)^ Furthermore, there is some evidence that appears to suggest extrafine formulations of ICS/LABA combinations provide better improvements in asthma control versus non-extrafine formulations.^(4,5,8)^ In this regard, the extrafine fixed combination of beclomethasone dipropionate (BDP) and formoterol fumarate (FF) represents the only extrafine combination in both pressurized metered-dose inhaler and dry powder inhalers (DPIs) developed until now.

Another key factor in successful asthma control is patient adherence, that is, not only using medication as prescribed but also using such treatment in the correct manner to maximize drug delivery.^(1,8)^ Patient adherence is impacted by multiple factors, including the type of device used to administer the medication.^(9)^

Given the importance of lung distribution in the efficacy of asthma drugs, a key factor in assessing ICS/LABA combinations is the adequate assessment of their lung deposition. The gold-standard technique for assessing lung deposition of inhaled drugs is *in vivo* scintigraphy.^(10,11)^ However, this technique requires radiolabeled products and exposure of the patient to radiation during the procedure. Various alternatives to scintigraphy exist, which use mathematical modeling to predict drug delivery and drug deposition in the airways. For example, functional respiratory imaging (FRI) combines three-dimensional (3D) lung models (obtained from high-resolution computerized tomography [HRCT] scans) with computational fluid dynamics (CFD). A validation study comparing CFD in CT-based airway models with combined single photon emission CT (SPECT)/CT in patients with mild asthma showed good agreement between the two techniques.^(12)^ Further investigations of FRI applied to chronic obstructive pulmonary disease (COPD) and asthma^(13–15)^ also demonstrated close agreement with scintigraphy results.^(16–23)^

The aim of our analyses was to compare the lung deposition in the extrathoracic, intrathoracic, and peripheral regions, using FRI, of ICS/LABA combinations in asthma patients when administered through three different and widely used DPIs: (1) Foster NEXThaler^®^, an extrafine formulation of BDP/FF, (2) Relvar Ellipta^®^, and (3) Symbicort Turbohaler^®^. As the inspiratory flow rate may influence the lung deposition of particles, which is of clinical importance in patients with severe asthma with usually low inspiratory flow rates, evaluations were conducted at different flow rates.

Key aims of our comparisons included the following: (1) to compare the lung deposition with NEXThaler versus Ellipta and Turbohaler and (2) to demonstrate flow independency of lung deposition with NEXThaler versus Turbohaler,

## Materials and Methods

We opted to use FRI, a technique that allows the study of aerosol deposition by coupling medical imaging to image processing and CFD.^(12)^ This methodology, which has been extensively described previously and validated,^(12,24,25)^ is based on four building blocks: (1) patient-specific 3D airway geometry modeling, (2) inhaler characteristics, (3) inhalation profile, and (4) CFD simulations to model lung deposition.

### Patient samples

The respiratory delivery of ICS and LABA from the three DPIs was investigated with patient-specific 3D airway geometry modeling using selected CT scans of 20 patients with asthma. The criteria for selecting patients were adults with asthma of various degrees of severity (Supplementary Table S1). An equal number of male and female patients were selected, with a wide range of age (median 54 years, range 26–73 years), height, and disease severity (median forced expiratory volume in 1 second [FEV_1_] 92.1% of predicted, range 50%–111.5%) (Supplementary Table S1).

All CT scans were generated in previous studies in which patient consent and approval from the relevant institutional review boards were procured; these analyses did not involve any active patient recruitment. The selected CT scans were accessed retrospectively. Informed consent was obtained from each patient for their scans to be used in these analyses, and ethical approval was granted by the Ethics Committee of the University Hospital in Antwerp.

### 3D airway modeling

For each selected patient, a scan was collected at the end of full inspiration (i.e., total lung capacity) and at the end of passive expiration (i.e., functional residual capacity). The particle simulations and visualizations were all performed on the total lung capacity scans. For comparing the different flow rates, the input parameters were the same for all devices and patients, that is, the inspired volumes were 1, 1.33, and 2.5 L for the 30, 40, and 60 L/min mean flow rates, respectively.

**FIG. 1.**
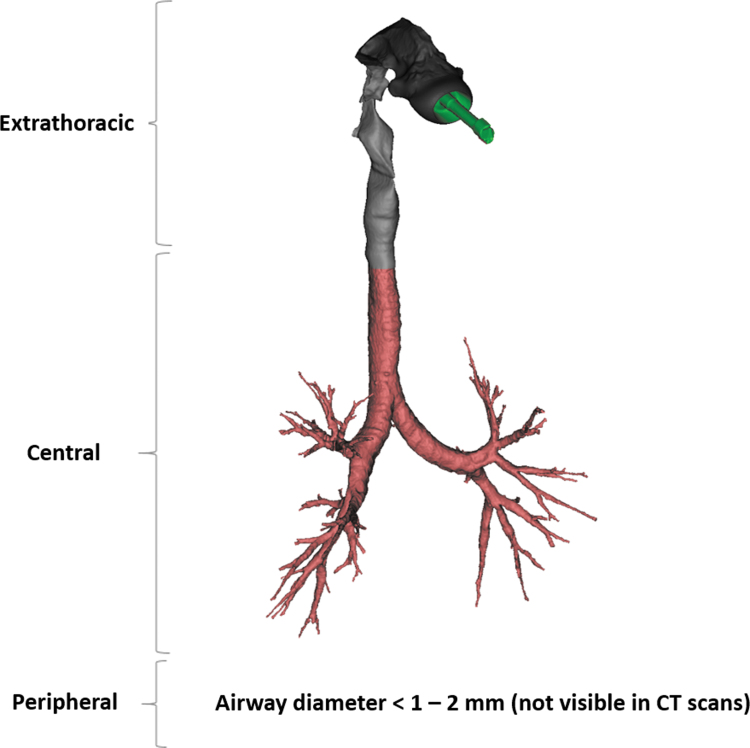
Patient-specific three-dimensional model of the upper and lower airways generated by high-resolution CT scans. CT, computerized tomography.

Patient-specific, volumetric, CT-based, 3D lung models were extracted from these scans to provide insights on the structural and functional characteristics of the respiratory system of each patient. The inspiratory scan was used to segment and model the patient-specific upper and lower airways to such an extent that the distinction between the intraluminal and alveolar airways was no longer feasible. This process translates into the reconstruction of the respiratory tract down to the level of the airways with a diameter of 1–2 mm, that is, corresponding to a CT scan voxel size of ∼0.5 mm^3^.^(26)^ The airways further downstream decrease in diameter size, thereby these airways cannot be distinguished in a CT scan. Hence, particles exiting the 3D model were considered to be deposited in the peripheral region. Nonetheless, by calculating the internal flow distribution, the involvement of this region can be accounted for. Therefore, the expiratory scan was used to measure the change in lobar volume from expiration to inspiration. Figure 1 shows an example of a 3D airway model from a representative asthma patient, in which the extrathoracic region (mouth and throat), the central (large and medium) airways, and the peripheral (small) airways of the respiratory tract are illustrated.

Commercially available validated software packages (Mimics 20.0 and 3-Matic 12.0, Materialise nv, Leuven, Belgium) were used for all segmentation and modeling operations.

### Inhaler characteristics

Foster NEXThaler DPI contains fixed-dose combinations of BDP and FF as extrafine particles and are approved for asthma (200 + 6 and 100 + 6 μg) and COPD (100 + 6 μg).^(27)^ The Relvar Ellipta DPI (92 + 22 μg fixed-dose combination of fluticasone furoate [FluF] and vilanterol trifenatate [Vil]) is licensed for asthma and COPD.^(28)^ Symbicort Turbohaler DPI (200 + 6 μg fixed-dose combination of budesonide [Bud] and FF) is also approved for asthma and COPD.^(29)^ According to Krüger et al.,^(30)^ who assessed inspiratory flow rates through marketed inhalers (three batches/product, three inhalers/batch, that is, nine inhalers/product), the inspiratory device resistance for NEXThaler, Ellipta, and Turbohaler was 0.036, 0.027, and 0.035 √kPa/(L/min), respectively. The inspiratory flow rate at 4.0 kPa pressure drop for NEXThaler, Ellipta, and Turbohaler was 54, 74, and 58 L/min, respectively.

For our analyses, the formulation characteristics of NEXThaler, Ellipta, and Turbohaler DPIs were obtained by high-performance liquid chromatography. The particle characteristics (mass median aerodynamic diameter [MMAD], geometrical standard deviation, fine particle fraction [FPF], and delivered dose [DD]) of the individual compounds (ICS and LABA) were measured using a cascade impactor. In addition, the drug release time from each device was provided. Particle characteristics for the DPIs evaluated are listed in Table 1.

**Table 1.** Particle Characteristics for NEXThaler DPI (BDP/FF), Ellipta DPI (FluF/Vil), and Turbohaler DPI (Bud/FF)

Device^a^	Active pharmaceutical ingredient	Mass median aerodynamic diameter (μm)	Geometric standard deviation	DD (μg)	Fine particle fraction (% of DD)	Drug release time (second)
60 L/min flow rate						
NEXThaler DPI 100 + 6 μg	BDP	1.3	2.6	81.9	56.8	0.099
	FF	1.5	1.8	5.0	58.7	
Ellipta DPI 92 + 22 μg	FluF	3.8	2.1	91.6	23.8	0.124
	Vil	2.1	2.1	22.5	45.3	
Turbohaler DPI 200 + 6 μg	Bud	2.1	1.8	163.5	62.2	0.137
	FF	2.1	1.9	4.6	62.8	
40 L/min flow rate						
NEXThaler DPI 100 + 6 μg	BDP	1.2	2.4	81.9	59.7	0.124
	FF	1.6	1.9	5.0	58.6	
Ellipta DPI 92 + 22 μg	FluF	4.1	2.1	90.7	23.7	0.131
	Vil	2.2	2.3	22.1	42.3	
Turbohaler DPI 200 + 6 μg	Bud	2.8	1.8	138.4	48.1	0.167
	FF	2.7	1.9	3.9	48.4	
30 L/min flow rate						
NEXThaler DPI 100 + 6 μg	BDP	1.5	2.6	84.4	57.3	0.171
	FF	2.3	2.0	4.9	54.6	
Turbohaler DPI 200 + 6 μg	Bud	3.0	1.9	125.8	29.5	0.407
	FF	2.9	1.8	3.5	29.8	

^a^ Doses shown are for ICS and LABA, respectively. Presented results are mean of two replicate measurements performed on a single unit for each device.

BDP, beclomethasone dipropionate; Bud, budesonide; DD, delivered dose; DPI, dry powder inhaler; FF, formoterol fumarate; FluF, fluticasone furoate; ICS, inhaled corticosteroid; LABA, long-acting beta_2_-agonist; Vil, vilanterol trifenatate.

### Inhalation profiles

Reverse-engineered CT scans of devices were virtually coupled to the mouth of the 3D models extracted from the CT lung scans. *In vitro* particle characteristics at three different mean flow rates (60, 40, and 30 L/min for NEXThaler and Turbohaler and 60 and 40 L/min for Ellipta) were measured to assess regional lung deposition patterns.

In the first instance, a DPI breathing profile, derived from the European Respiratory Society/International Society for Aerosols in Medicine (ERS/ISAM) Task Force,^(31)^ was created for a fast inhalation with a duration of 2.5 seconds to achieve a mean flow rate of 60 L/min (Supplementary Fig. S1). Two other DPI breathing profiles were also assessed, that is, inhalations lasting 2.0 seconds to achieve mean flow rates of ∼40 L/min and ∼30 L/min (Supplementary Fig. S1). NEXThaler and Turbohaler were assessed at 60, 40, and 30 L/min mean flow rates. Ellipta was only assessed at 60 and 40 L/min mean flow rates.

### CFD simulations

The 3D models of the respiratory tracts were divided into two global regions: extrathoracic (from the mouth up to the upper airways) and intrathoracic (from around the sternum up to the airways further downstream). The intrathoracic region was further subdivided into the central airways (diameter >1 or 2 mm and visible on a CT scan; that is, large and medium airways) and the peripheral (small) airways, which were not visible on the CT scan (Fig. 1).

Triangulated surface meshes created in 3-Matic (Materialise NV) were converted to tetrahedral 3D volume meshes using TGrid 14.0 (Ansys, Inc., Canonsburg, PA). Subsequently, CFD simulations were performed on the 3D models, taking into account the following boundary conditions: the inhalation profile was applied at the inlet of the inhaler to account for flow turbulence generated by the device; the percentage of flow exiting the model toward a lobe was equal to the relative lobar expansion as obtained from patient-specific inspiratory and expiratory lobar 3D models; although some particles are exhaled, the particles not deposited in either the extrathoracic or central (large and medium) airways were considered to be deposited in the peripheral airways; and no-slip conditions were chosen for the airway walls, that is, particles were trapped when hitting the wall. The mathematical model and appropriate boundary conditions have been previously validated for flow distribution, but not for aerosol deposition predictions.^(12)^

### Data analyses

The modeled deposition data in the global lung regions (extrathoracic deposition, intrathoracic deposition, and peripheral deposition) and the lobar lung regions as a percentage of the nominal dose are described descriptively, that is, mean ± standard deviation. Differences in intrathoracic and extrathoracic deposition between NEXThaler (comparator) and Ellipta, and Turbohaler were analyzed using a linear regression. For the global lung region data, the central-to-peripheral (C/P) deposition ratio, which is a measure to define the distribution of intrathoracic deposition over the larger and smaller airways, was calculated by dividing the deposition in the central airways over the deposition in the peripheral airways.

## Results

### Patients

**FIG. 2.**
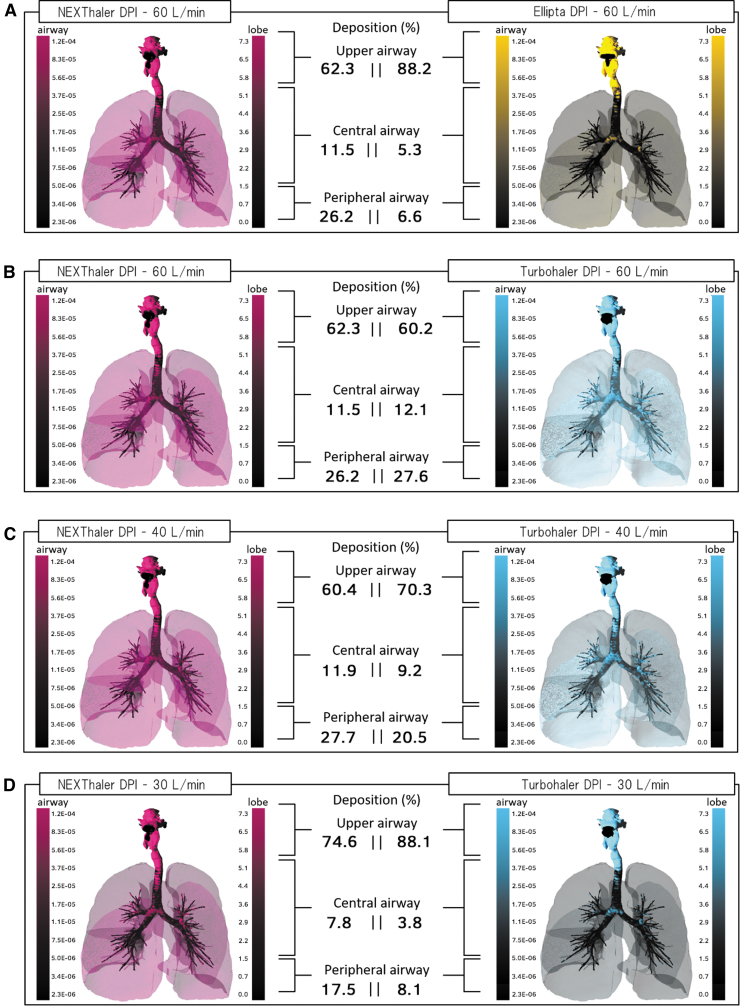
Representative modeled deposition of **(A)** NEXThaler versus Ellipta at 60 L/min, and NEXThaler versus Turbohaler at **(B)** 60, **(C)** 40, and **(D)** 30 L/min in one patient. DPI, dry powder inhaler. Lighter areas correspond to higher regional deposition concentrations.

The individual characteristics of the 20 selected patients in our study are reported in Supplementary Table S1. The modeled deposition in the lungs and airways following modeling for NEXThaler, Ellipta, and Turbohaler is depicted in one representative patient in Figure 2.

### NEXThaler versus Ellipta

**FIG. 3.**
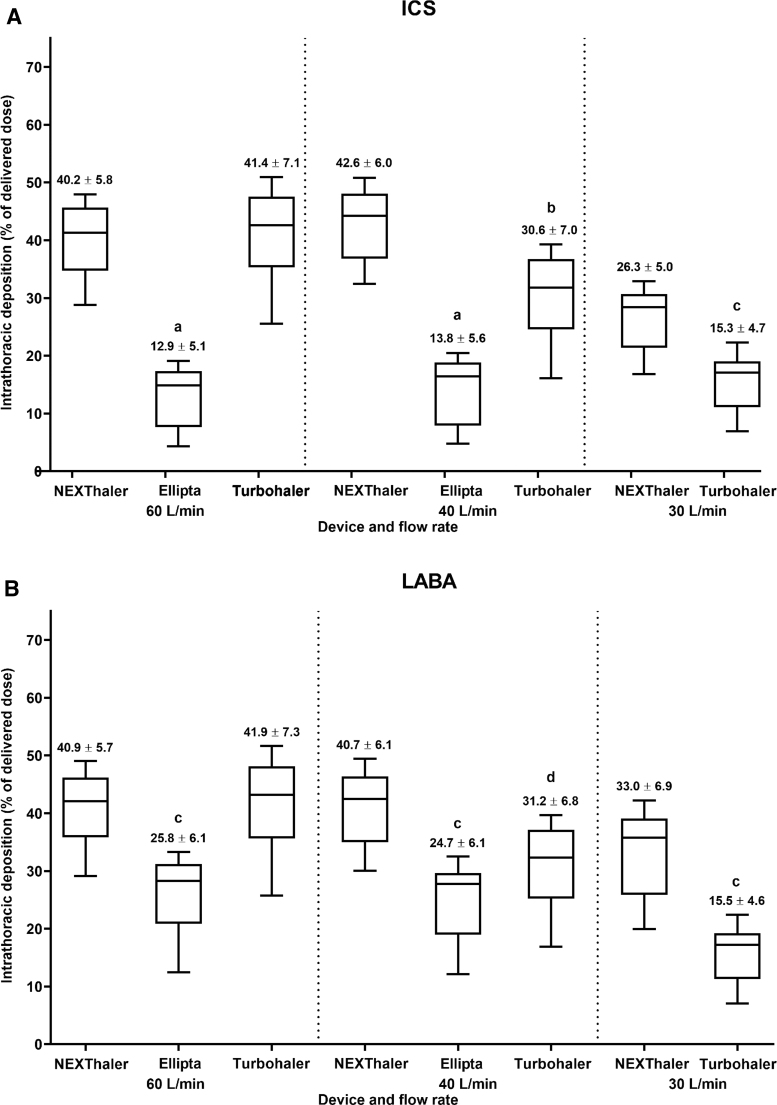
Intrathoracic deposition for **(A)** ICS and **(B)** LABA components for NEXThaler (BDP/FF; at 60, 40, and 30 L/min), Ellipta (FluF/Vil; at 60 and 40 L/min), and Turbohaler (Bud/FF; at 60, 40 and 30 L/min). Box and whisker plots show minimum 25th quartile, median, 75th quartile, and maximum values. Data above each bar are mean ± SD. ^a^*p* < 0.005, ^b^*p* < 0.01, ^c^*p* < 0.001, and ^d^*p* < 0.05 (linear regression analysis) versus NEXThaler. Doses of ICS and LABA, respectively, for each device are 100 + 6 μg (NEXThaler), 99 + 22 μg (Ellipta), and 200 + 6 μg (Turbohaler). BDP, beclomethasone dipropionate; Bud, budesonide; FF, formoterol fumarate; FluF, fluticasone furoate; ICS, inhaled corticosteroid; LABA, long-acting beta_2_-agonist; SD, standard deviation; Vil, vilanterol trifenatate.

Lung deposition with Ellipta DPI was only modeled at 60 and 40 L/min mean flow rates. The mean intrathoracic deposition was significantly higher with NEXThaler compared with Ellipta both for ICS (*p* < 0.001) and LABA (*p* < 0.005) at both 60 and 40 L/min mean flow rates (Fig. 3; individual patient data are reported in Supplementary Tables S2 and S3). The intrathoracic delivery of ICS and LABA components was similar to NEXThaler, whereas the ICS intrathoracic delivery was lower than LABA lung deposition with Ellipta (Fig. 3).

**FIG. 4.**
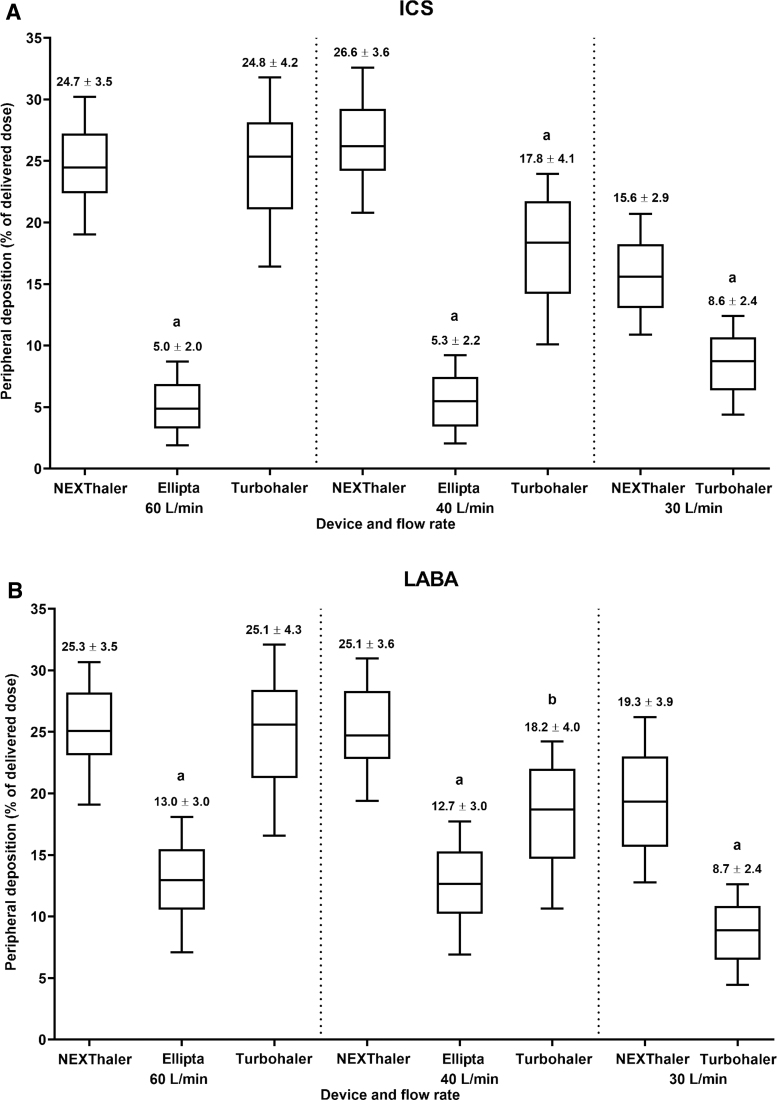
Peripheral deposition for ICS and LABA components for NEXThaler (BDP/FF; at 60, 40 and 30 L/min), Ellipta (FluF/Vil; at 60 and 40 L/min), and Turbohaler (Bud/FF; at 60, 40 and 30 L/min). Box and whisker plots show minimum 25th quartile, median, 75th quartile, and maximum values. Data above each bar are mean ± SD. ^a^*p* < 0.001 and ^b^*p* < 0.005 (linear regression analysis) versus NEXThaler. Doses of ICS and LABA, respectively, for each device are 100 + 6 μg (NEXThaler), 99 + 22 μg (Ellipta), and 200 + 6 μg (Turbohaler).

At 60 and 40 L/min flow rates, mean peripheral deposition of ICS and LABA were ∼5x and ∼2x higher, respectively, with NEXThaler compared with Ellipta (*p* < 0.001 and *p* < 0.005; Fig. 4; individual patient data are shown in Supplementary Tables S2 and S3). The peripheral delivery of both ICS and LABA components was similar with NEXThaler, whereas ICS peripheral delivery was lower than LABA lung deposition with Ellipta (Fig. 4).

**FIG. 5.**
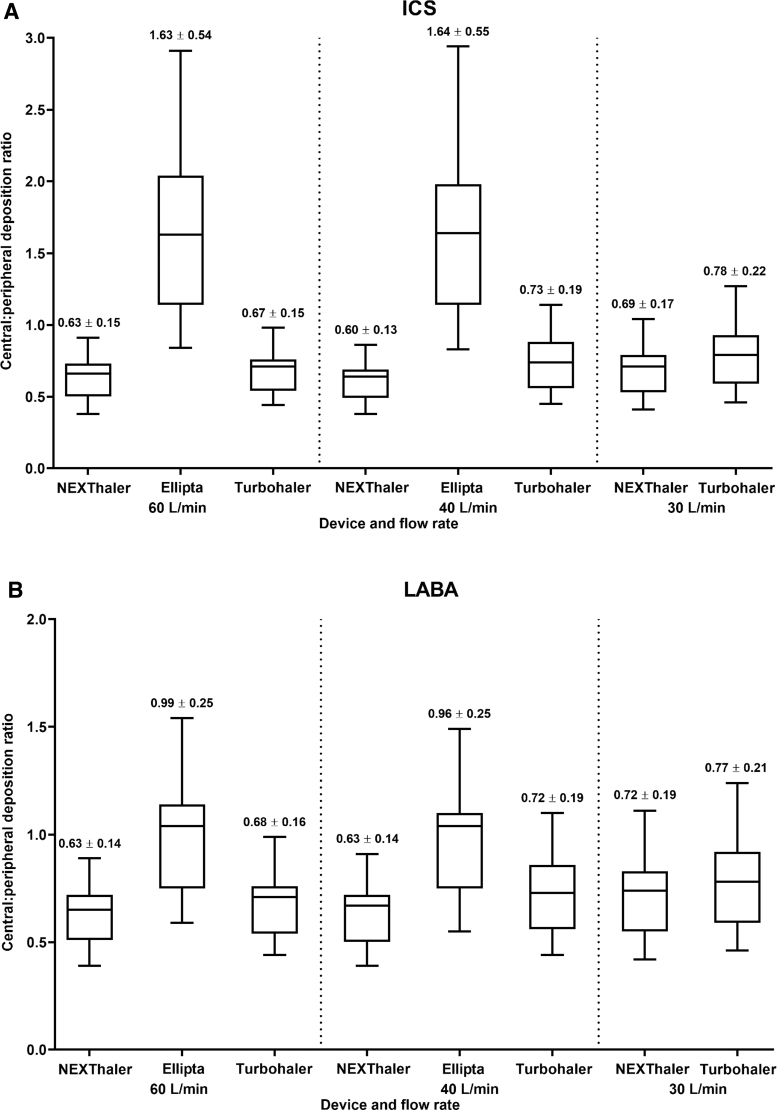
Central:peripheral deposition for ICS and LABA components for NEXThaler (BDP/FF; at 60, 40, and 30 L/min), Ellipta (FluF/Vil; at 60 and 40 30 L/min), and Turbohaler (Bud/FF; at 60, 40, and 30 L/min). Box and whisker plots show minimum 25th quartile, median, 75th quartile, and maximum values. Data above each bar are mean ± SD. Doses of ICS and LABA, respectively, for each device are 100 + 6 μg (NEXThaler), 99 + 22 μg (Ellipta), and 200 + 6 μg (Turbohaler).

At both flow rates tested (60 and 40 L/min), the mean C/P ratio with NEXThaler was lower (that is, representing higher peripheral deposition) than with Ellipta for both ICS and LABA (Fig. 5; individual patient data are shown in Supplementary Tables S2 and S3). Notably, the two components of BDP/FF administered with NEXThaler showed a similar C/P distribution pattern (regardless of flow rate), whereas the C/P distribution pattern with Ellipta showed a lower peripheral deposition (higher C/P ratio) of FluF than Vil (regardless of flow rate) (Fig. 5).

### NEXThaler versus Turbohaler

Lung deposition with the Turbohaler DPI was modeled at 60, 40, and 30 L/min mean flow rates. Mean intrathoracic deposition with NEXThaler was similar to Turbohaler for both ICS and LABA at 60 L/min (Fig. 3; individual patient data are shown in Supplementary Tables S2 and S4). In contrast, NEXThaler achieved significantly higher mean intrathoracic depositions of both active ingredients versus Turbohaler at both 40 (*p* < 0.001 and *p* < 0.005) and 30 (*p* < 0.001) L/min flow rates; lung deposition by Turbohaler at 40 and 30 L/min flow rates was ∼26% and ∼47% lower, respectively, compared with NEXThaler (Fig. 3).

At 60 L/min flow rate, mean peripheral deposition of the active ingredients was similar with NEXThaler (ICS: 24.7%; LABA: 25.3%) and Turbohaler (ICS: 24.8%; LABA: 25.1%) (Fig. 4; individual patient data are shown in Supplementary Tables S2 and S4). Mean peripheral lung deposition of ICS and LABA was significantly lower with Turbohaler compared with NEXThaler at 40 (*p* < 0.01 and *p* < 0.05) and 30 (*p* < 0.001) L/min flow rates, by ∼30% and ∼50%, respectively (Fig. 4).

At 60, 40, and 30 L/min flow rates, the C/P ratios were similar with NEXThaler for both active components (Fig. 5; individual patient data are shown in Supplementary Table S2). Both components of Bud/FF administered with Turbohaler showed a slight increase in C/P distribution pattern (that is, lower peripheral deposition) with lower flow rates of 40 and 30 L/min compared with 60 L/min flow rates (Fig. 5; individual patient data are shown in Supplementary Table S4).

## Discussion

Our aim was to model (using FRI) the lung deposition in the extrathoracic, intrathoracic, and peripheral regions of ICS and LABA dispensed from three DPIs commonly used for asthma control, thereby allowing a comparison between these devices, with particular emphasis on peripheral lung deposition and the impact of inhalation flow rates on lung deposition. For the peripheral lung, NEXThaler (extrafine formulation of BDP/FF delivered by a DPI) had a higher lung deposition of both ICS and LABA, and a consistent C/P ratio for both active ingredients, compared with values for Ellipta; this was irrespective of flow rate. Moreover, lung deposition of ICS and LABA was less impacted by flow rate with NEXThaler than with Turbohaler.

The particle characteristics for the three devices evaluated in our study are generally comparable with previously published data for both ICS and LABA components; that is, DD and FPF for NEXThaler and Turbohaler at 60, 40, and 30 L/min flow rates^(32)^; MMAD and FPF at 60 L/min for all three DPIs^(33)^; and DD for Ellipta at 60 and 40 L/min,^(34)^ thus confirming the validity of our lung deposition evaluations of these DPIs.

The intrathoracic deposition of both ICS and LABA with NEXThaler was greater than that with Ellipta regardless of the flow rate. This observation is likely due to the higher FPF of NEXThaler versus Ellipta at both flow rates evaluated. In contrast, the intrathoracic deposition of both active ingredients was similar with NEXThaler and Turbohaler DPIs at 60 L/min flow rate, despite a slightly lower FPF with NEXThaler compared with the Turbohaler. However, intrathoracic deposition of ICS and LABA was affected by the flow rate for the Turbohaler, as there were large decreases in FPF at 40 L/min (∼25%) and 30 L/min (∼50%) compared with 60 L/min flow rate, resulting in decreases in deposition in this clinically important lung region, which is consistent with previous reports.^(35)^ Indeed, in a study of 20 asthma patients given Bud/FF by either Z7200 device or Turbohaler DPI, FRI showed increased airway volume for all patients using Z7200, although an increase was not seen with Turbohaler in three patients.^(36)^ A possible explanation for this difference is that the particle characteristics of Turbohaler are affected by breathing pattern. Thus, for patients who are unable to generate the optimal breathing profile, there may be a large drop in lung deposition with the Turbohaler. The FPF of ICS and LABA for NEXThaler was comparable at all flow rates evaluated, although the intrathoracic deposition at 30 L/min was lower than at 40 and 60 L/min. Nevertheless, the lung deposition results with NEXThaler at 30 L/min are important as a flow rate of 35 L/min is the minimum required to activate the mechanism in this DPI.^(37)^ Our findings showing the positive ICS and LABA lung deposition from NEXThaler, even at flow rates of 30 and 40 L/min, are consistent with previous observations showing that the NEXThaler breath-actuated mechanism can be activated by all patients with asthma, even those with severe disease.^(37)^

In view of the large proportion of patients with asthma who are inadequately controlled with current treatments,^(3)^ targeting the peripheral lung, and at patient-achievable flow rates, is a key treatment goal in asthma, particularly as small airway dysfunction is known to be involved in asthma control across all severities of the disease.^(4–7)^ Hence, our findings that the peripheral lung deposition of ICS and LABA being higher with NEXThaler compared with Ellipta DPI and comparable with the Turbohaler DPI at 60 L/min flow rate may be of clinical relevance. Our findings are likely due to the peripheral lung deposition being impacted by particle size, that is, MMAD.^(35,38)^

A study using a method that generated monodisperse aerosols with different particle sizes (1.5-, 3-, and 6.5-μm MMAD) of albuterol, in 12 asthma patients, demonstrated that smaller particles had more peripheral lung deposition (25%, 17%, and 10%, respectively); however, larger particles achieved a greater change in FEV_1_.^(38)^ In contrast, an earlier study that tested different particle sizes of terbutaline generated from a nebulizer (eterodisperse aerosols) found that there was no difference in lung function parameters between large and small particles for FEV_1_, forced vital capacity (FVC), and peak expiratory flow (PEF), but small particles were more effective on maximal flow after expiration of 50% of FVC (Vmax_50_) and maximal flow after expiration of 75% of FVC (Vmax_25_).^(39)^ Furthermore, in 754 asthma patients, extrafine NEXThaler DPI was noninferior to extrafine BDP/FF administered through a pressurized metered-dose inhaler for the primary endpoint (change from baseline for the entire 8-week treatment period in average predose morning peak expiratory flow), and both of these treatments were statistically superior to a nonextrafine BDP DPI.^(40)^ Indeed, NEXThaler is the only available DPI that delivers extrafine particles (MMAD <2 μm), shown to be the appropriate size required to reach the peripheral airways (which typically have an internal diameter <2 mm).^(37)^ The MMAD for particles emitted from the Ellipta DPI was higher than with NEXThaler and Turbohaler DPIs for both ICS and LABA; moreover, higher MMAD values occurred at lower flow rates. Indeed, the FRI modeling demonstrated that such differences in MMADs affected peripheral lung deposition of ICS and LABA delivered by the DPIs investigated.

For the peripheral lung, NEXThaler had a higher deposition of both ICS and LABA compared with Ellipta regardless of the flow rate (40 or 60 L/min). By contrast, peripheral lung deposition of ICS and LABA was less impacted by flow rate with NEXThaler compared with the Turbohaler.

The differences in intrathoracic and peripheral lung depositions described above affected the C/P ratios. The C/P ratios for Ellipta were higher for ICS (1.63–1.64) and LABA (0.96–0.99) than NEXThaler (0.63–0.69 and 0.63–0.72, respectively) and Turbohaler (0.67–0.79 and 0.68–0.77, respectively). Furthermore, in contrast to Ellipta, this ratio was similar for ICS and LABA with NEXThaler DPI and Turbohaler DPI, indicating consistent lung deposition of both active ingredients with these two devices. Our findings on the pattern of lung deposition with DPIs are broadly consistent with previous reports that show smaller inhaled particles have more peripheral lung deposition.^(35,38)^ As we did not apply a normalization factor in calculating the C/P ratio, it must be recognized that a C/P ratio <1 may still result in a higher local dose of an active ingredient (that is, number of deposited particles/airway surface area) in the central airways region than in the peripheral airways, given the much larger surface area of the latter. However, we are using the C/P ratio to compare the deposition of ICS and LABA in a similar region between the different devices, and not to provide an exact indication on how the inhalation devices will behave in a real-life setting.

The FRI procedure examining key differences in lung deposition between DPIs has several benefits. First, this technique has lower radiation exposure and costs compared with SPECT/CT. Importantly, FRI has been validated versus SPECT/CT in patients,^(12)^ with several investigations showing acceptable agreement between these methods.^(13–23)^ Another strength of this technique is it allows for modeling and direct comparison of the characteristics of DPIs at different inhalation flow rates in the same set of scans from patients with asthma. However, one limitation of our study is that scans from only 20 patients were used in our evaluations. A further limitation was that it was not possible to distinguish between particles depositing in the small airways and the alveolar region in the 3D model simulations. In addition, since particles not deposited in either the extrathoracic or central (large and medium) airways were considered to be deposited in the peripheral airways, it is possible that this could have led to some overestimation of the fraction of deposited aerosols in the peripheral airways. Nevertheless, our results with NEXThaler using FRI are in close agreement with previous data examining the lung deposition (including the periphery) of ICS/LABA with NEXThaler in healthy volunteers, and patients with asthma and COPD using a gamma-scintigraphy technique,^(41)^ supporting the validity of our findings.

## Conclusions

Our FRI deposition modeling data demonstrate key differences between NEXThaler, Ellipta, and Turbohaler DPIs in the lung deposition of ICS and LABA. NEXThaler DPI showed better lung deposition (higher intrathoracic deposition due to a larger FPF), particularly in the peripheral lung (due to lower MMAD values), compared with Ellipta DPI, irrespective of flow rate. Moreover, lung deposition was relatively independent of flow rate with NEXThaler, in contrast with Turbohaler that showed markedly lower deposition at lower flow rates. Thus, DPI administration of an extrafine formulation is more efficient than larger particle formulations in delivering ICS and LABA consistently not only to the central airways but also to the peripheral airways in asthma. These advantages of NEXThaler could potentially result in improved clinical outcomes in patients with asthma.^(42–44)^

## Supplementary Material

Supplemental data

Supplemental data

Supplemental data

Supplemental data

Supplemental data

## Data Availability

Study data will be made available from Chiesi Farmaceutici in response to any reasonable request.

## Notes

Reviewed by:

Chantal Darquenne

Nicola Scichilone
