# Supplementary material for: Targeting the Small Airways with Inhaled Corticosteroid/Long-Acting Beta Agonist Dry Powder Inhalers: A Functional Respiratory Imaging Study
Source: J Aerosol Med Pulm Drug Deliv. 2021 Sep 27;34(5):280–92. doi: 10.1089/jamp.2020.1618 (PMC8573800; doi:10.1089/jamp.2020.1618)
Supplement: Supplemental data [file Supp_TableS1.docx]

**SUPPLEMENTARY TABLE S1**. Individual patient characteristics

| **Patient** | **Sex** | **Age (years)** | **Height (cm)** | **FEV_1_ (% of predicted)** |
| --- | --- | --- | --- | --- |
| 1 | Female | 60 | 161.9 | 116 |
| 2 | Female | 25 | 172.2 | 52 |
| 3 | Female | 26 | 172.4 | 50 |
| 4 | Male | 46 | 180.0 | 107.8 |
| 5 | Female | 59 | 170.1 | 95 |
| 6 | Female | 73 | 152.9 | 111.5 |
| 7 | Female | 56 | 161.0 | 106.3 |
| 8 | Female | 46 | 168.0 | 92.8 |
| 9 | Male | 63 | 177.0 | 64.8 |
| 10 | Male | 37 | 175.0 | 93.9 |
| 11 | Male | 29 | 176.0 | 90.3 |
| 12 | Male | 45 | 169.5 | 107.6 |
| 13 | Male | 66 | 174.0 | 80.6 |
| 14 | Male | 54 | 177.5 | 87.4 |
| 15 | Female | 55 | 162.0 | 100.5 |
| 16 | Male | 63 | 169.0 | 62.5 |
| 17 | Female | 50 | 162.0 | 80.1 |
| 18 | Male | 54 | 176.0 | 117.6 |
| 19 | Female | 58 | 163.0 | 91.6 |
| 20 | Male | 26 | 176.0 | 79.3 |
| **Median (range)** |  | **54**  **(26–73)** | **171.2**  **(152.9–180.0)** | **92.2**  **(50–111.5)** |

FEV_1_, forced expiratory volume
