# Supplementary figures and images for: Targeting the Small Airways with Inhaled Corticosteroid/Long-Acting Beta Agonist Dry Powder Inhalers: A Functional Respiratory Imaging Study
Source: J Aerosol Med Pulm Drug Deliv. 2021 Sep 27;34(5):280–92. doi: 10.1089/jamp.2020.1618 (PMC8573800; doi:10.1089/jamp.2020.1618)

**SUPPLEMENTARY FIG. S1.** Breathing profiles at flow rates of 30, 40 and 60 L/min.
DPI, dry powder inhaler.


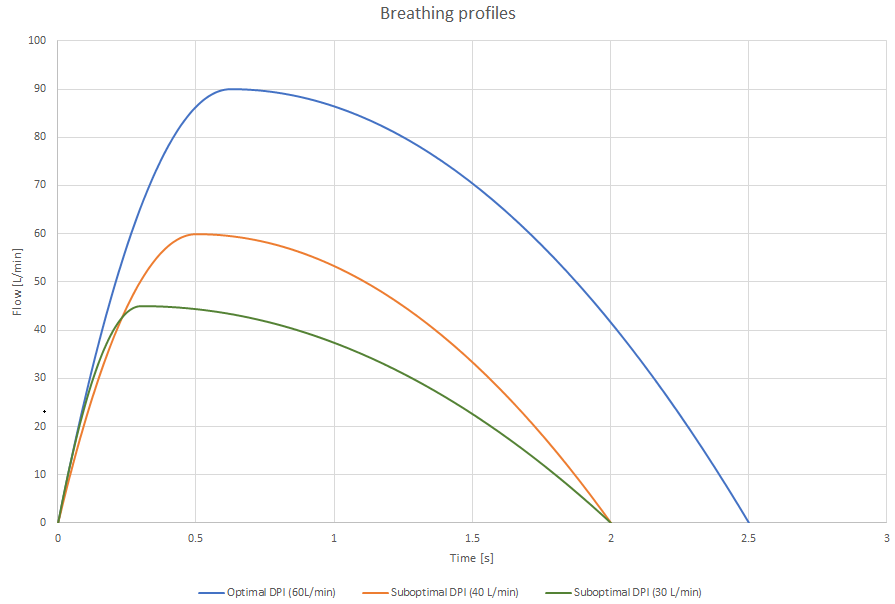

Supplement: Supplemental data [file Supp_FigS1.docx]
