# Supplementary material for: Targeting the Small Airways with Inhaled Corticosteroid/Long-Acting Beta Agonist Dry Powder Inhalers: A Functional Respiratory Imaging Study
Source: J Aerosol Med Pulm Drug Deliv. 2021 Sep 27;34(5):280–92. doi: 10.1089/jamp.2020.1618 (PMC8573800; doi:10.1089/jamp.2020.1618)
Supplement: Supplemental data [file Supp_TableS2.docx]

**SUPPLEMENTARY TABLE S2**. Modelled lung deposition for NEXThaler DPI (BDP/FF; 100/6 µg) in the global lung regions for 60 L/min flow rate: individual patient characteristics

| **Patient** | **Deposition (% of nominal dose)** | | | | | | | | **Ratio** | |
| --- | --- | --- | --- | --- | --- | --- | --- | --- | --- | --- |
|  | **Extrathoracic** | | **Intrathoracic** | | **Central** | | **Peripheral** | | **C/P** | |
|  | BDP | FF | BDP | FF | BDP | FF | BDP | FF | BDP | FF |
| 1 | 54.3 | 53.6 | 45.7 | 46.4 | 15.7 | 15.7 | 29.9 | 30.7 | 0.53 | 0.51 |
| 2 | 64.9 | 64.0 | 34.9 | 35.8 | 9.6 | 10.0 | 25.3 | 25.8 | 0.38 | 0.39 |
| 3 | 65.3 | 64.7 | 34.7 | 35.3 | 14.7 | 15.1 | 20.0 | 20.1 | 0.73 | 0.75 |
| 4 | 71.2 | 70.9 | 28.8 | 29.1 | 9.8 | 10.0 | 19.0 | 19.1 | 0.51 | 0.52 |
| 5 | 65.5 | 64.2 | 34.4 | 35.7 | 11.4 | 11.9 | 23.0 | 23.8 | 0.50 | 0.50 |
| 6 | 58.0 | 57.2 | 41.9 | 42.8 | 17.8 | 17.9 | 24.2 | 25.0 | 0.73 | 0.72 |
| 7 | 57.0 | 56.5 | 43.0 | 43.5 | 13.3 | 13.5 | 29.7 | 30.0 | 0.45 | 0.45 |
| 8 | 54.3 | 54.3 | 45.6 | 45.6 | 20.8 | 20.5 | 24.8 | 25.2 | 0.84 | 0.81 |
| 9 | 64.2 | 63.0 | 35.8 | 37.0 | 11.9 | 12.4 | 24.0 | 24.6 | 0.50 | 0.50 |
| 10 | 52.0 | 50.9 | 47.9 | 49.0 | 20.4 | 20.4 | 27.6 | 28.6 | 0.74 | 0.71 |
| 11 | 65.4 | 64.0 | 34.5 | 35.9 | 13.9 | 14.5 | 20.7 | 21.4 | 0.67 | 0.68 |
| 12 | 53.8 | 53.2 | 46.1 | 46.8 | 21.3 | 21.3 | 24.8 | 25.4 | 0.86 | 0.84 |
| 13 | 56.0 | 54.9 | 44.0 | 45.1 | 17.8 | 18.1 | 26.2 | 27.0 | 0.68 | 0.67 |
| 14 | 62.3 | 61.8 | 37.6 | 38.1 | 11.5 | 11.5 | 26.1 | 26.6 | 0.44 | 0.43 |
| 15 | 52.7 | 52.5 | 47.3 | 47.4 | 17.9 | 17.2 | 29.4 | 30.2 | 0.61 | 0.57 |
| 16 | 59.2 | 58.6 | 40.7 | 41.3 | 16.6 | 16.7 | 24.1 | 24.6 | 0.69 | 0.68 |
| 17 | 67.0 | 66.3 | 32.9 | 33.7 | 13.3 | 13.6 | 19.7 | 20.1 | 0.674 | 0.68 |
| 18 | 52.3 | 51.8 | 47.7 | 48.1 | 17.5 | 17.5 | 30.2 | 30.6 | 0.58 | 0.57 |
| 19 | 57.0 | 56.5 | 43.0 | 43.5 | 20.5 | 20.53 | 22.5 | 23.0 | 0.91 | 0.89 |
| 20 | 63.1 | 61.4 | 36.8 | 38.5 | 14.4 | 15.0 | 22.4 | 23.5 | 0.64 | 0.64 |
| **Mean**  **(SD)** | **59.8**  **(5.9)** | **59.0**  **(5.7)** | **40.2**  **(5.8)** | **40.9**  **(5.7)** | **15.5**  **(3.7)** | **15.7**  **(3.5)** | **24.7**  **(3.5)** | **25.3**  **(3.5)** | **0.63**  **(0.15)** | **0.63**  **(0.14)** |

BDP, beclometasone dipropionate; C/P, central:peripheral ratio; DPI, dry powder inhaler; FF, formoterol fumarate; SD, standard deviation.
