# Supplementary material for: Targeting the Small Airways with Inhaled Corticosteroid/Long-Acting Beta Agonist Dry Powder Inhalers: A Functional Respiratory Imaging Study
Source: J Aerosol Med Pulm Drug Deliv. 2021 Sep 27;34(5):280–92. doi: 10.1089/jamp.2020.1618 (PMC8573800; doi:10.1089/jamp.2020.1618)
Supplement: Supplemental data [file Supp_TableS3.docx]

**SUPPLEMENTARY TABLE S3**. Modelled lung deposition for Ellipta DPI (FluF/Vil) in the global lung regions for 60 L/min flow rate: individual patient characteristics

| **Patient** | **Deposition (% of nominal dose)** | | | | | | | | **Ratio** | |
| --- | --- | --- | --- | --- | --- | --- | --- | --- | --- | --- |
|  | **Extrathoracic** | | **Intrathoracic** | | **Central** | | **Peripheral** | | **C/P** | |
|  | FluF | Vil | FluF | Vil | FluF | Vil | FluF | Vil | FluF | Vil |
| 1 | 81.5 | 67.5 | 18.5 | 32.5 | 9.8 | 14.4 | 8.7 | 18.1 | 1.13 | 0.80 |
| 2 | 93.0 | 79.6 | 7.0 | 20.4 | 3.2 | 7.5 | 3.8 | 12.8 | 0.84 | 0.59 |
| 3 | 90.5 | 78.1 | 9.5 | 21.9 | 6.4 | 11.7 | 3.1 | 10.2 | 2.08 | 1.15 |
| 4 | 95.7 | 87.5 | 4.3 | 12.5 | 2.4 | 5.4 | 1.9 | 7.1 | 1.28 | 0.76 |
| 5 | 93.2 | 81.0 | 6.8 | 19.0 | 3.6 | 8.0 | 3.2 | 10.9 | 1.11 | 0.74 |
| 6 | 83.3 | 68.6 | 16.7 | 31.4 | 10.7 | 16.2 | 6.0 | 15.2 | 1.78 | 1.07 |
| 7 | 84.0 | 69.4 | 16.0 | 30.6 | 8.2 | 12.7 | 7.8 | 17.9 | 1.05 | 0.71 |
| 8 | 81.0 | 67.2 | 19.0 | 32.8 | 13.1 | 18.1 | 5.8 | 14.7 | 2.25 | 1.23 |
| 9 | 92.9 | 79.2 | 7.1 | 20.8 | 3.8 | 8.9 | 3.3 | 11.9 | 1.17 | 0.75 |
| 10 | 80.9 | 66.7 | 19.1 | 33.3 | 11.8 | 17.7 | 7.3 | 15.6 | 1.63 | 1.14 |
| 11 | 93.1 | 80.7 | 6.9 | 19.3 | 4.6 | 10.2 | 2.4 | 9.1 | 1.94 | 1.12 |
| 12 | 83.3 | 71.2 | 16.7 | 28.8 | 11.4 | 16.5 | 5.3 | 12.3 | 2.15 | 1.34 |
| 13 | 82.4 | 69.6 | 17.5 | 30.3 | 11.8 | 16.1 | 5.7 | 14.2 | 2.07 | 1.14 |
| 14 | 90.5 | 78.6 | 9.5 | 21.4 | 4.5 | 8.3 | 4.9 | 13.1 | 0.92 | 0.63 |
| 15 | 81.2 | 67.1 | 18.8 | 32.9 | 11.6 | 16.1 | 7.2 | 16.8 | 1.62 | 0.95 |
| 16 | 86.0 | 72.0 | 14.0 | 28.0 | 9.2 | 14.8 | 4.8 | 13.2 | 1.94 | 1.12 |
| 17 | 90.6 | 79.0 | 9.4 | 21.0 | 6.2 | 11.2 | 3.2 | 9.8 | 1.89 | 1.14 |
| 18 | 83.8 | 70.0 | 16.2 | 30.0 | 8.9 | 14.3 | 7.3 | 15.8 | 1.21 | 0.91 |
| 19 | 84.2 | 71.4 | 15.8 | 28.5 | 11.7 | 17.3 | 4.0 | 11.2 | 2.91 | 1.54 |
| 20 | 90.7 | 79.0 | 9.3 | 21.0 | 5.8 | 10.6 | 3.5 | 10.4 | 1.64 | 1.02 |
| **Mean**  **(SD)** | **87.1**  **(5.1)** | **74.2**  **(6.2)** | **12.9**  **(5.1)** | **25.8**  **(6.1)** | **7.9**  **(3.5)** | **12.8**  **(3.9)** | **5.0**  **(2.0)** | **13.0**  **(3.0)** | **1.63**  **(0.54)** | **0.99**  **(0.25)** |

C/P, central:peripheral ratio; DPI, dry powder inhaler; FluF, fluticasone furoate; SD, standard deviation; Vil, vilanterol trifenatate.
