# Supplementary material for: Targeting the Small Airways with Inhaled Corticosteroid/Long-Acting Beta Agonist Dry Powder Inhalers: A Functional Respiratory Imaging Study
Source: J Aerosol Med Pulm Drug Deliv. 2021 Sep 27;34(5):280–92. doi: 10.1089/jamp.2020.1618 (PMC8573800; doi:10.1089/jamp.2020.1618)
Supplement: Supplemental data [file Supp_TableS4.docx]

**SUPPLEMENTARY TABLE S4**. Modelled lung deposition for Turbohaler DPI (Bud/FF) in the global lung regions for 60 L/min flow rate: individual patient characteristics

| **Patient** | **Deposition (% of nominal dose)** | | | | | | | | **Ratio** | |
| --- | --- | --- | --- | --- | --- | --- | --- | --- | --- | --- |
|  | **Extrathoracic** | | **Intrathoracic** | | **Central** | | **Peripheral** | | **C/P** | |
|  | Bud | FF | Bud | FF | Bud | FF | Bud | FF | Bud | FF |
| 1 | 53.2 | 52.5 | 46.8 | 47.4 | 16.7 | 17.0 | 30.1 | 30.4 | 0.56 | 0.56 |
| 2 | 66.3 | 66.0 | 33.6 | 33.9 | 10.2 | 10.3 | 23.4 | 23.6 | 0.44 | 0.44 |
| 3 | 63.5 | 63.1 | 36.4 | 36.8 | 16.3 | 16.5 | 20.1 | 20.3 | 0.81 | 0.81 |
| 4 | 74.5 | 74.2 | 25.5 | 25.8 | 9.1 | 9.2 | 16.4 | 16.58 | 0.55 | 0.55 |
| 5 | 59.9 | 59.5 | 40.1 | 40.5 | 13.8 | 14.0 | 26.3 | 26.57 | 0.52 | 0.53 |
| 6 | 52.6 | 52.0 | 47.4 | 48.0 | 20.3 | 20.7 | 27.05 | 27.3 | 0.75 | 0.76 |
| 7 | 55.8 | 55.2 | 44.2 | 44.8 | 14.8 | 15.0 | 29.47 | 29.8 | 0.50 | 0.50 |
| 8 | 50.7 | 50.0 | 49.2 | 50.0 | 22.6 | 23.1 | 26.65 | 26.9 | 0.85 | 0.86 |
| 9 | 68.0 | 67.7 | 31.9 | 32.3 | 11.1 | 11.2 | 20.8 | 21.0 | 0.53 | 0.53 |
| 10 | 50.6 | 49.9 | 49.4 | 50.1 | 21.1 | 21.5 | 28.3 | 28.6 | 0.75 | 0.75 |
| 11 | 66.8 | 66.4 | 33.2 | 33.5 | 14.0 | 14.2 | 19.2 | 19.4 | 0.73 | 0.73 |
| 12 | 53.7 | 53.1 | 46.3 | 46.9 | 21.9 | 22.3 | 24.4 | 24.6 | 0.90 | 0.91 |
| 13 | 52.4 | 51.8 | 47.6 | 48.2 | 20.5 | 20.9 | 27.1 | 27.3 | 0.76 | 0.76 |
| 14 | 60.2 | 59.9 | 39.7 | 40.1 | 12.1 | 12.3 | 27.6 | 27.8 | 0.44 | 0.44 |
| 15 | 49.1 | 48.4 | 50.9 | 51.6 | 19.1 | 19.5 | 31.8 | 32.1 | 0.60 | 0.61 |
| 16 | 58.9 | 58.4 | 41.1 | 41.6 | 17.5 | 17.8 | 23.6 | 23.8 | 0.74 | 0.75 |
| 17 | 65.1 | 64.7 | 34.9 | 35.3 | 15.1 | 15.2 | 19.9 | 20.0 | 0.76 | 0.76 |
| 18 | 52.4 | 51.8 | 47.5 | 48.1 | 17.8 | 18.1 | 29.7 | 30.1 | 0.60 | 0.60 |
| 19 | 54.5 | 54.0 | 45.4 | 46.0 | 22.5 | 22.9 | 22.9 | 23.1 | 0.98 | 0.99 |
| 20 | 63.4 | 63.0 | 36.6 | 37.0 | 14.9 | 15.0 | 21.7 | 21.9 | 0.68 | 0.69 |
| **Mean**  **(SD)** | **58.6**  **(7.1)** | **58.1**  **(7.3)** | **41.4**  **(7.1)** | **41.0**  **(7.3)** | **16.6**  **(4.2)** | **16.8**  **(4.3)** | **24.8**  **(4.2)** | **25.1**  **(4.3)** | **0.67**  **(0.15)** | **0.68**  **(0.16)** |

Bud, budesonide; C/P, central:peripheral ratio; DPI, dry powder inhaler; FF, formoterol fumarate; SD, standard deviation.
